# Posture and Texting: Effect on Balance in Young Adults

**DOI:** 10.1371/journal.pone.0134230

**Published:** 2015-07-31

**Authors:** Nurul Retno Nurwulan, Bernard C. Jiang, Hardianto Iridiastadi

**Affiliations:** 1 Department of Industrial Management, School of Management, National Taiwan University and Science and Technology, Taipei, Taiwan, Republic of China; 2 Department of Industrial Engineering, Faculty of Industrial Technology, Institut Teknologi Bandung, Bandung, Indonesia; West Virginia University, UNITED STATES

## Abstract

Using a mobile phone while doing another activity is a common dual-task activity in our daily lives. This study examined the effect of texting on the postural stability of young adults. Twenty college students were asked to perform static and dynamic postural stability tasks. Traditional COP and multivariate multiscale entropy (MMSE) were used to assess the static postural stability and the Star Excursion Balance Test (SEBT) was used to assess the dynamic postural stability. Results showed that (1) texting impaired postural stability, (2) the complexity index did not change much although the task conditions changed, and (3) performing texting is perceived to be more difficult.

## Introduction

Using a mobile phone while doing another activity is a common example of dual-task activities that we often do just about anywhere. The use of mobiles phone by pedestrians while walking has an impact on working memory [1] and increases walking distractions that put pedestrians at higher risk for accidents [2, 3, 4]. Pedestrians’ behaviors are considered to be one of the causes of pedestrians’ injuries, because the data from police often do not mention any drivers’ mistakes [5].

The cognitive distraction from using a mobile phone reduces situation awareness and increases unsafe behavior, such as ignoring traffic lights and not looking left and right while crossing the road [2, 5]. Distracted pedestrians are less likely to successfully cross the road when they are talking on the phone [3, 4]. A study by Schwebel et al. [6] found that listening to music and texting are more distracting than talking on the phone, because texting involves reading and typing: an activity which is more cognitively demanding than talking, while listening to music is a constant auditory disturbance [6]. These previous studies examined the distracting effect of using mobile phones. However, they only analyzed the behavioral effect of using mobile phones that may cause accidents.

Previous postural stability studies mostly used traditional COP method as their means of measurement. However, human gait and posture are considered to be a dynamic, complex, and non-linear process. Entropy-based methods have been considered to be a better measurement for analyzing the center of pressure (COP) of balance and gait due to their ability to measure the uncertainty of non-linear dynamic systems. Multiscale entropy (MSE) has been proven to be an effective method in evaluating signal complexities over different time scales [7]. However, MSE can only consider data channels separately, which is only appropriate if the multivariate signals are independent and there is no correlation statistically [8]. To overcome this problem, Ahmed and Mandic [8] proposed multivariate multiscale entropy (MMSE). This method is very promising for analyzing postural stability, due to its sensitivity to changes and its ability to distinguish different sways more clearly [9, 10].

Based on the fact that previous studies regarding the distracting effects of mobile mostly only analyze the behavioral effect, more thorough examination is needed to quantify its effect by postural stability analysis. Further, previous postural stability studies mostly only used traditional COP, which is not really suitable for human postural data. It seems clear that there is a need to do more evaluation regarding the effect of using mobile phones on postural stability. Thus, the purpose of this study was to evaluate the effect of texting on postural stability using MMSE. The participants of this study were college students, because almost all college students use mobile phone with great frequency [4] and believe texting is the most appropriate method of communication in all circumstances [11]. This study used the traditional COP and MMSE methods in order to rigorously evaluate the effect of secondary tasks on postural stability, including not only the effect of secondary tasks on posture but also the underlying effects of complex dynamical behavior from the environment.

## Methods

### Participants

The participants in this study were 20 college students (mean: 21.75 ± 1.59 years, Table 1), recruited from National Taiwan University of Science and Technology. All participants were free of orthopedic and neurological disorders based on self-reports. The experiment was approved by the Institutional Review Board, Department of Health, Executive Yuan, R.O.C (Taiwan). All participants signed informed consent forms before participating in this study. The individual in this manuscript has given written informed consent (as outlined in PLOS consent form) to publish these case details.

**Table 1:** Participants Demographic Details.

No	Sex	Age	Height (cm)	Weight (kg)	Waist (cm)	Hip (cm)	Leg (cm)
1	Male	20	172	57	76	89	99
2	Male	24	172	69	83.5	93.5	83
3	Male	22	185	61	76	88	95
4	Male	22	170	54	78	93	90
5	Male	22	160	58	75	89	72
6	Male	20	180	68	79	92	91
7	Male	21	168	78	88	102	92
8	Male	23	169	55	64	76	85
9	Male	22	167	64.2	73	94	79
10	Male	20	176	55	80	86	95
11	Female	22	162.5	47	70	90	79
12	Female	24	159	45	70	85	73
13	Female	20	169	53	75	95	96
14	Female	24	156	45	64	83	85
15	Female	20	158	58	73	94	90
16	Female	21	170	55	67	96	92
17	Female	22	163	50	75	87	90
18	Female	25	158	47	67	75	79
19	Female	20	165	55	74	97	93
20	Female	21	160	67	70	89	92

### Apparatus

AMTI force platform model OR6–7 was used to assess static postural stability and the Star Excursion Balance Test (SEBT) was used to assess dynamic postural stability. The COP data were exported and computed by Matlab version 7.13.0.564 [12]link12 for further analysis. A subjective rating scale and Mackworth Clock Test (MCT) were used to measure participants’ cognitive loads.

### Procedures

**Fig 1:**
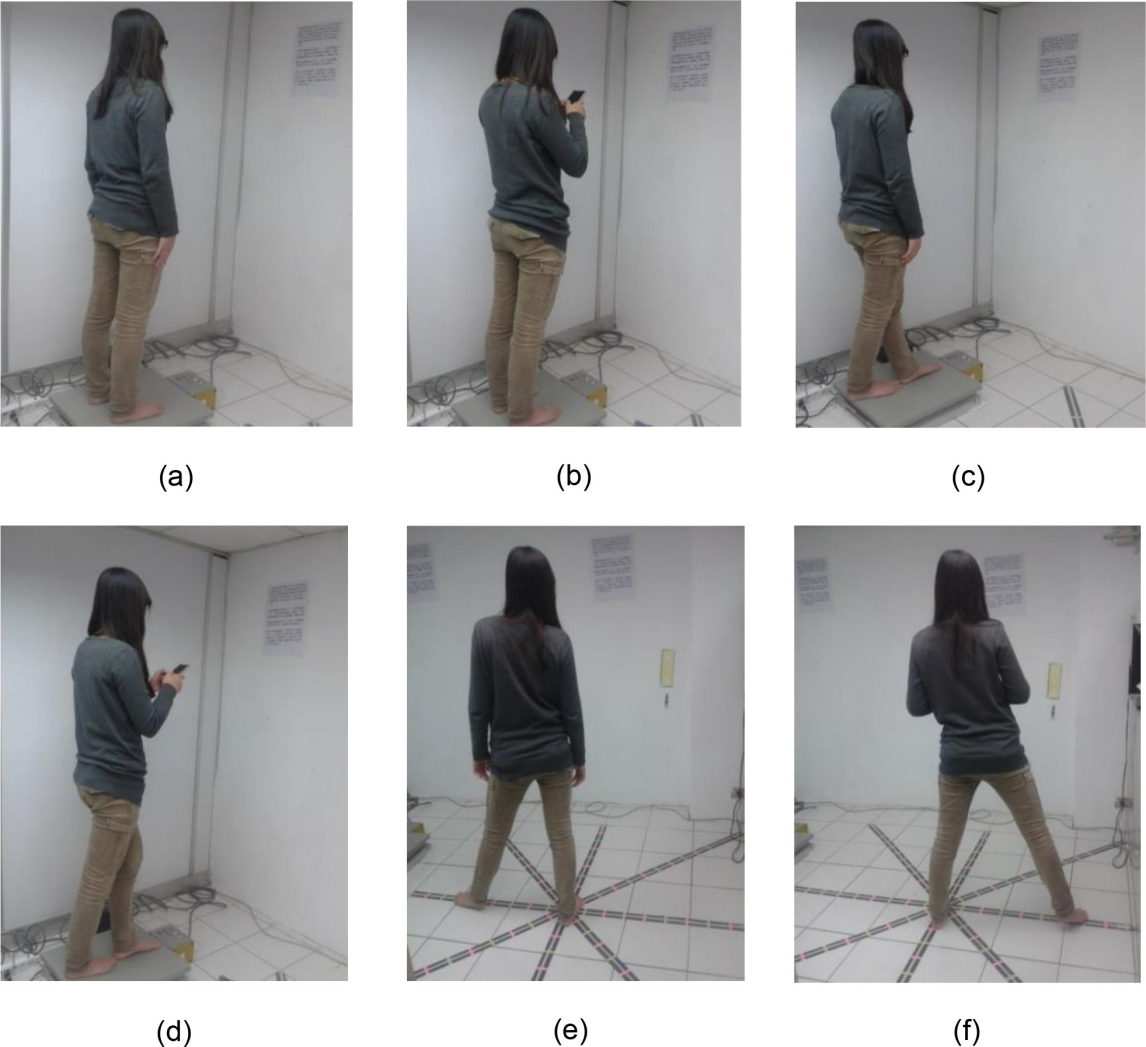
Task Conditions. The subjects stood barefoot, performing six different task conditions for 65 seconds: (a) normal stance, (b) normal stance and texting, (c) tandem stance (heal-to-toe), (d) tandem stance and texting, and (e) star excursion balance test, (f) star excursion balance test and texting.

For static postural stability (Fig 1), the participants stood barefoot on a force platform for 65 seconds (the first 5 seconds data were eliminated), performing 4 different task conditions: normal stance, normal stance with texting, tandem stance (Romberg test), and tandem stance with texting. For dynamic postural stability (Fig 1), participants performed the SEBT task without and with texting. The texting content was put right in front of the participants. The texting content was one paragraph of an article, made in Chinese and English versions because some participants were international students. It is assumed that this difference did not cause any significant difference, because the purpose of the texting was to give some distraction to the participants.

The participants performed 3 trials of each task and were allowed to take 3-minutes break between trials and 10-minutes breaks before moving to a different task condition in order to assess the cognitive load. The participants performed the MCT for 5 minutes. In the subjective rating scale, participants were asked to rate their mental efforts (very low mental effort, low mental effort, neither low nor high mental effort, high mental effort, very high mental effort) and task difficulties (very easy, easy, neither easy nor difficult, difficult, very difficult) of each task.

The COP data were computed by Matlab to analyze the traditional COP and MMSE. For the SEBT data, the reach distances were normalized by dividing it by the leg lengths. Mean response time was used to analyze the MCT in order to examine participants’ cognitive loads. Paired t-test was used to compare the COP and the SEBT data between tasks. Wilcoxon test was used to analyze the subjective rating scale and MCT data.

### Multivariate Multiscale Entropy

Postural stability has been studied in various ways with a range of different measures. The most typical measure of postural stability is Center of Pressure (COP) because it can be obtained from a force platform directly [13]. However, the output signals from the human body are dynamic, non-linear and non-stationary [14].

Since the MSE method was proposed by Costa et al. [14], it has been effectively applied in physiological, biological, and geoscientifical data analyses [7]. However, the algorithm was designed for scalar time series analysis and not suited for multivariate time series such as experimental and biological systems [8]. Furthermore, MSE has bias problem when the scales increase and it is not well-adapted to nonlinear and non-stationary signals with a low sampling rate.

The MMSE evaluates multivariate sample entropy (MSampEn) over different time scales in rigorous and unified ways to cater for both within- and cross-channel correlations in multiple data channels [8]. The MMSE is considered to be better than MSE due to its sensitivity in detecting the distinct complexity of postural stability [9]. Previous studies showed that MMSE is a very promising method for analyzing postural stability [9, 10].

## Results

In general, postural stability performance deteriorated while texting. In the case of static postural stability, the sway was greater in a texting condition. In the case of dynamic postural stability, reaching distance of the SEBT was shorter during a texting condition.

### Static Postural Stability

Mean distance, total excursion, mean displacement velocity, and sway area were used to analyze the results based on traditional COP. The study showed significant differences between conditions with and without texting in all stabilogrametric parameters of the traditional COP. Participants exhibited more sway in all parameters during texting condition, supporting the idea that a secondary task impairs postural stability.

**Fig 2:**
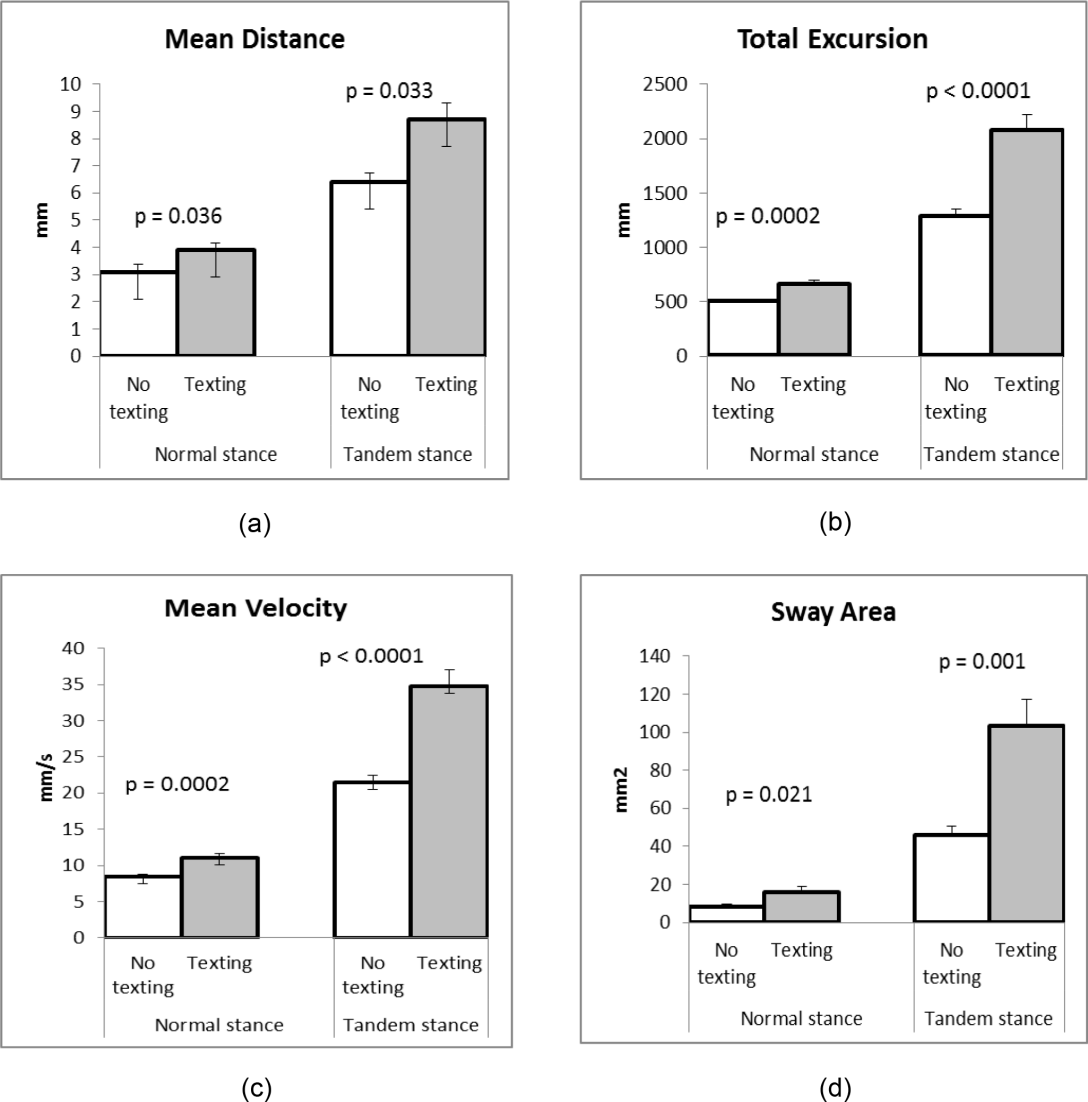
Mean ± SE values of stabilogrametric parameters of traditional COP. Mean and standard error values of traditional COP parameters: (a) mean distance between situations with and without texting for normal stance and tandem stance are significantly different (p = 0.036 and p = 0.033, respectively), (b) total excursion between situations with and without texting for normal stance and tandem stance are significantly different (p = 0.0002 and p ≤ 0.0001, respectively), (c) mean velocity between situations with and without texting for normal stance and tandem stance are significantly different (p = 0.0002 and p ≤ 0.0001, respectively), and (d) sway area between situations with and without texting for normal stance and tandem stance are significantly different (p = 0.021 and p = 0.001, respectively).

Regarding the fact that 3 participants lost their balances during the experiment, the data of these participants were excluded from the analysis. One participant was not cooperating and the other two participants lost their balance for unknown reasons. The paired t-test showed significant differences between situations with and without texting for both stances in mean distances (p = 0.036, p = 0.033 normal stance and tandem stance respectively, Fig 2) were found. Total excursion and mean velocity increased when participants performed texting task in both stances. There were significant differences between situations with and without texting in regards to normal stance and tandem stance (p = 0.0002, p < 0.0001, Fig 2).

**Fig 3:**
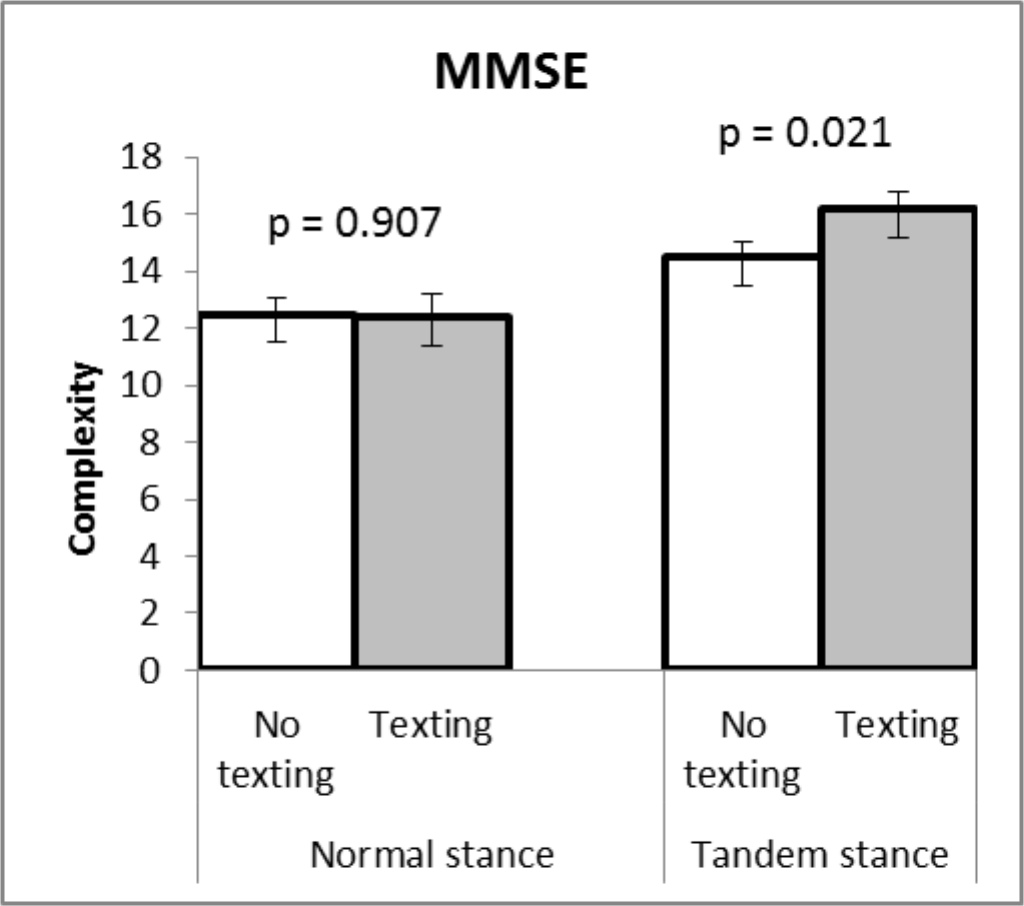
Mean ± SE values of MMSE. Mean and standard error values of multivariate multiscale entropy (MMSE): no difference between situations with and without texting in normal stance (p = 0.907), but significant difference between situations with and without texting in tandem stance (p = 0.021).

The sway area were bigger while texting. The differences between situations with and without texting for both stances were significant (p = 0.021, p = 0.001 for normal stance and tandem stance respectively, Fig 2). For the MMSE analysis, the only significant difference (p = 0.021, Fig 3) occurred in situations with and without texting in the tandem stance.

### Dynamic Postural Stability

To assess dynamic postural stability, the subjects performed the SEBT without and with texting for 60 seconds. The reaching distances were divided by leg lengths and then multiplied by 100 [15]. Then, eight directions of normalized reaching distance were summed up, because the reaching directions do not significantly affect the performance of SEBT [16]. The paired t-tests between the SEBT without texting (mean 509.26 ± 59.68) and the SEBT with texting (420.33 ± 70.59) were significantly different (p < 0.0001).

### Cognitive Load

Cognitive load, based on task differences can significantly affect the degree of vigilance decrement [17]. To assess the cognitive load of the dual-task activity, a Mackworth Clock Test (MCT) and subjective rating scale were used. The MCT was originally created to examine vigilance during the prolonged visual searches of radar operators during World War II [18]. A single clock hand moved in equal increments around the clock face, with the exception of occasional larger jumps. In this test, participants were asked to report when they detected the larger jumps. Vigilance decrement is known when the participants showed a decline in signal detection over time.

Mean response times of the MCT were significantly different between situations with and without texting for both stances (p = 0.025 and p = 0.004 for normal stance and tandem stance, respectively). This suggests that performing a postural task and texting create a bigger cognitive load for the participants than when there is an absence of texting.

For the subjective rating scale, there were differences between situations with and without texting in all postural tasks in terms of mental effort and task difficulty. There were differences in mental effort between with and without texting in a normal stance (p = 0.007), a tandem stance (p = 0.014), and with SEBT (p = 0.0003). Differences in task difficulty between situations with and without texting in a normal stance (p = 0.008), a tandem stance (p = 0.004), and SEBT (p = 0.00028) were also found. These findings showed performing dual-task activity was perceived to be difficult by the participants.

## Discussion

A previous study by Schabrun et al. [19] investigated the impact of texting on gait performance. They found texting modified gait performance and could negatively impact the balance system. However, they did not evaluate the impact of texting on postural stability while the subjects were standing still. This study is the first to compare the effect of texting on static postural stability using traditional COP and MMSE. The mean distance, total excursion, mean velocity, and sway area were used as traditional COP methods.

The mean distance is the vector distance from the mean COP to the point in *y*_*0*_ (*n*) and *x*_*0*_ (*n*), which represents the average distance from the mean COP [20]. This study found texting increased mean distance, which shows that the participants swayed more when performing standing tasks with texting. The significant differences between situations with and without texting for both stances showed that performing postural tasks with texting resulted in longer average distances from the mean COP. The difference in mean distances between normal and tandem stances in this study was in agreement with a previous study [21] that found the mean distance of young adults increased from 3% to 15% when performed harder postures.

The total excursion is the total length of the COP path and is obtained by summing up the distances of the consecutive points on the COP path [20]. Significant differences between situations with and without texting in a normal stance and a tandem stance show that the participants need to increase the length of the COP path to maintain balance while performing dual-tasks. This result is consistent with previous studies [22, 23] which found secondary task increased total excursion.

The mean displacement velocity is the average velocity of the COP [20], it is obtained from dividing total excursion by the experimental time. A large difference between normal and tandem stances indicates the more difficult postural task demands made on participants in order for them to keep stability while moving at a faster speed. Teasdale and Simoneau [24] found different postural conditions affected mean velocity. The result of this study is consistent with the findings of Yang [23] who found significant differences between situations with and without secondary tasks in young adults (p = 0.001).

Sway area is defined as the area enclosed by the COP path per unit of time. It can be conceptualized as proportional to the product of mean distance and mean velocity. Sway area can be obtained by summing the area of the triangles formed by two consecutive points on the COP path and the mean COP [25]. This study found significant differences between situations with and without texting in both stances. Another dual-task study by Yang [23] also found significant differences between situations with and without secondary task in young adults (p = 0.005). Measurements of sway while performing dual-tasks indicated that the participants became more unstable and more likely to fall.

The traditional COP method could detect the effect of secondary tasks on postural stability. However, it could not analyze the factors underlying poorer stability. The multiscale entropy (MSE) method was introduced because human physiological data are dynamic, non-linear, and non-stationary [14]. Complexity is used in analyzing the physiological data in MSE analysis. Complexity is a biologic system which reflects the ability to adapt and function in a changing environment [26]. Healthy physiological systems are often characterized by an irregular and complex type of variability. Disease or aging is often associated with greater regularity and less complexity [27].

A number of previous studies have compared young-elderly participants or healthy-sick participants [7–11, 23, 27]. Higher complexity indicates better adaptability to an external environment: the higher the complexity index the better the postural stability [9, 23, 27]. However, this study found that performing dual-tasks created higher complexity. This might be because past studies compared young and older adults, or healthy participants and participants with illnesses. Therefore, the young and healthy subjects are expected to exhibit high complexity, whereas the elderly and sick participants are expected to have low complexity. In this study, all participants were young and healthy. The study indicates that when considering young and healthy participants as the baseline of a stable system, they have a better ability to adjust to challenges from the external environment in order to keep postural stability.

The MMSE was proposed to overcome the limitations of MSE. It is able to evaluate the structural complexity of multivariate systems, obtained by measuring the relative complexity of the multichannel signals through the plot of the multivariate sample entropy [8]. The significant difference between with and without texting was only found in the tandem stance. This indicates that although young adults have good adaptability to respond to unexpected internal and external disruptions regarding task conditions and to anticipate changes during the tasks, performing secondary tasks while in rather complex postures could be dangerous for balance. This study showed not only the distracting effect of secondary tasks on posture, but also the effects of complex dynamic behavior that underlie the causes of changes in balance.

For the SEBT, the total reaching distance decreased 17.46%. The MCT was used to assess the psychological effect of performing dual-task activity, while the subjective rating scale was used to assess the psychophysical effect. The significant difference of mean response times between situations with and without texting was found. This result indicated that texting while doing tandem stance posture creates bigger cognitive load to the participants.

Texting is the most frequent communication method for college students. It is considered beneficial for self-esteem [28], and college students feel anxious and lonely when they cannot communicate with their friends by texting [29]. This psychological issue might be the reason why college students use mobile phones while doing other activities most of the time. The significant differences between situations with and without texting in all of postural task conditions showed that performing dual-task activity is perceived to be difficult and need high mental efforts. College students feel the urge to use their phones all the times, although they realize it is difficult to do. Therefore, college students are very vulnerable to get injured because of using mobile phones while doing other activities.

## Conclusion

This study is the first to compare the effect of texting on static postural stability using traditional COP and MMSE. In this study, the comparison between situations with and without texting in 3 different postural tasks (normal stance, tandem stance, and the SEBT) showed significant differences. The participants perceived that performing dual-task activity was difficult. The significant differences between situations with and without texting in all postural tasks in terms of mental effort and task difficulty were found. However, the complexity index did not change much although the task conditions changed. The perturbations from the task conditions did not affect the complexity index. The participants were able to anticipate and respond to the unexpected changes from the task conditions because younger adults have good adaptability to keep their balances and keep on functioning in order to prevent falling. Taken together, this study has shown that using mobile phones impair postural stability of the college students. In the future research, similar study can be used to examine the effect of using mobile phone on postural stability of older adults, since the behavior of younger adults and older adults in using mobile phone are different. For deeper analysis, future research should compare the results of younger adults and older adults, in order to know how aging can affect the distracting effect of using mobile phones.

## Supporting Information

S1 File Experiment Data. (RAR)
